# An overview of control strategy and diagnostic technology for foot-and-mouth disease in China

**DOI:** 10.1186/1743-422X-10-78

**Published:** 2013-03-07

**Authors:** Yao-Zhong Ding, Hao-Tai Chen, Jie Zhang, Jian-Hua Zhou, Li-Na Ma, Liang Zhang, Yuanxin Gu, Yong-Sheng Liu

**Affiliations:** 1 State Key Laboratory of Veterinary Etiological Biology, OIE/China National Foot-and-Mouth Disease Reference Laboratory, Lanzhou Veterinary Research Institute, Chinese Academy of Agricultural Sciences, Lanzhou 730046, China

**Keywords:** Foot-and-mouth disease, Control strategy, Chinese veterinary administrative system for FMD, Diagnostic technique

## Abstract

Foot-and-mouth disease (FMD) is one of most contagious animal diseases. It affects millions of cloven-hoofed animals and causes huge economic losses in many countries of the world. There are seven serotypes of which three (O, A and Asia 1) are endemic in China. Efficient control of FMD in China is crucial for the prevention and control of FMD in Asia and throughout the world. For the control of FMD, a powerful veterinary administration, a well-trained veterinary staff, a system of rapid and accurate diagnostic procedures and, in many countries, compulsory vaccination of susceptible animals are indispensable. This article strives to outline the Chinese animal disease control and prevention system, in particular for FMD, with the emphasis on diagnostic procedures applied in Chinese laboratories. In addition, new technologies for FMD diagnosis, which are currently in the phase of development or in the process of validation in Chinese laboratories, are described, such as lateral flow devices (LFD), Mab-based ELISAs, reverse transcription loop-mediated isothermal amplification (RT-LAMP) and gold nanopariticle immuno-PCR (GNP-IPCR).

## Introduction

Foot-and-mouth disease (FMD) is one of the most contagious diseases of mammals which has caused severe economic losses for production of susceptible cloven-hoofed animals. There are seven serotypes of FMD virus (FMDV), namely, O, A, C, SAT 1, SAT 2, SAT 3 and Asia 1. The genome of the virus is over 8 kb in length and encodes four structural proteins (SPs, VP1, VP2, VP3 and VP4) that form an icosahedral capsid [1], and a total of ten mature non-structural proteins (NSPs) (L, 2A, 2B, 2C, 3A, 3B, 3C, 3D, 3AB or 3ABC).

This disease has been controlled successfully in many countries with the strategies such as mandatory vaccination of susceptible animals and slaughtering of infected animals. However, no country has been considered safe due to the highly contagious nature of the virus and the intensified international trade of animal or animal products [2]. FMD serotypes O, Asia 1 and A have been endemic in different regions in China since 1999. To control the spread of the disease and to eradicate the virus, many measures have been taken by the Chinese government including compulsory immunisation, an animal disease reporting system, the development of rapid diagnostic techniques and the generation of a new vaccine.

In this article, we introduced the FMD control and prevention system in China, routine diagnostic methods in OIE/China National Foot-and-Mouth Disease Reference Laboratory and some new methods, which are still not validated for FMD diagnosis, such as LFD, IPCR and LAMP.

### FMD control and prevention system

The Standing Committee of the National People’s Congress or veterinary administrative department of Ministry of Agriculture in China proclaimed several laws for the purpose of FMD control such as the Animal Epidemic Prevention Law of the People’s Republic of China, The Quarantine Law of the People’s Republic of China on the Import and Export Animal and Plant, Emergency Measures for Handling Major Animal Epidemics and Contingency plans of Prevention and control of Foot-and-mouth Disease.

The organization for FMD control and prevention consists of four levels of veterinary administrative departments ranging from central, provincial, and municipal to the county government, an OIE/China National Foot-and-mouth Disease reference laboratory, animal disease monitoring stations and border animal epidemic monitoring stations.

### Foot and mouth disease reporting procedures

(A) Foot and Mouth Disease suspected case report procedure: The suspected case was first confirmed when one of the clinical signs or syndromes of foot-and-mouth disease appears on susceptible animals in accordance with epidemiology characteristics of FMD. According to the law, the veterinarians or farmers should promptly report the FMD suspected case to the local Animal Disease Control and Prevention Center (ADC). At least 2 official veterinarians in the ADC of that county should immediately go to the holdings to check and collect samples of the suspected FMD case and subsequently take control measures after receiving the report. The confirmed suspected FMD cases should be reported to the local veterinary bureau and the higher administrative authority. The samples of FMD suspected cases should be collected and sent to the provincial animal disease control center, or to the national foot-and-mouth disease reference laboratory, if necessary. The official veterinarian should take necessary control measures for FMD suspected cases, including separation and monitoring of the suspected FMD case, movement restriction of animal or animal products, and disinfection of the holding and the environment before the laboratory diagnostic result was obtained.

(B) Confirmed FMD report procedure: The outbreak of FMD can be confirmed if the result for one of the FMDV causative agent assays in laboratory diagnosis was positive or 3ABC nonstructural protein was positive for the FMD suspect case. The provincial ADC should report the FMD case within 1 hour to the provincial veterinary administrative department and Center for Animal Disease Control and Prevention (CADC) of the People’s Republic of China. The provincial veterinary administrative department should report the FMD confirmed case to the government of the province and the national veterinary administrative department within 1 hour.

(C) OIE/China National Foot-and-mouth Disease Reference Laboratory should report the diagnostic result to national CADC and the veterinary competent authority of the state council of China, and notify, at the same time, the provincial veterinary administrative department, as well as the provincial CADC, of the outbreak of FMD.

(D) The veterinary competent authority of the state council of China is responsible for declaring the confirmed FMD outbreak to the OIE base on the laboratory diagnostic results conducted by the provincial animal disease control center or the national foot-and-mouth reference laboratory and making sure that the necessary control measures to eradicate an FMD epidemic are taken as soon as possible.

### The main FMD control measures in China

In China, a compulsory vaccination strategy for FMD is applied. The costs of vaccination are jointly funded by the central and provincial governments. Inactivated or peptide vaccines for FMDV are produced by different manufacturers which all have to be licensed by the Ministry of Agriculture. The quality of FMD vaccines are controlled by the Chinese Institute for the Control of Veterinary Drugs. The annual compulsory vaccination plan is approved by the Ministry of Agriculture. In recent years, a compulsory vaccination scheme has been followed which includes immunization of all pigs against serotype O (with inactivated or peptide vaccines) and all cattle, sheep, goat, deer and camel against serotypes O and Asia 1 (with inactivated vaccines), while all dairy cows and bulls as well as susceptible animals in border areas are also immunized against serotype A (with inactivated vaccines). The interval of FMDV vaccine immunization is no more than six months. The regular serological survey should ensure that the qualified rate of titer of FMDV antibody in the herd reached more than 70%. It is necessary to prevent any spread of the disease as soon as an outbreak occurs by monitoring livestock movements and emergency booster vaccination. When an FMD outbreak is confirmed, the control measures are as follows.

Firstly, infected and contaminated susceptible animals should be slaughtered without delay. Animal bodies and suspected contaminated feces, litter, feed and sewage should be disinfected under official supervision in accordance with the instructions provided by the official veterinarian. Then, the strict disinfection of contaminated traffic vehicles, animal houses and relevant tools should be conducted. Thirdly, the local ADC should trace animals dispatched from the infected zones during the period of at least 21 days before the estimated date of earliest infection to monitor the spread of disease. Next, the veterinary administrative departments delimit the warning signs around the epidemic area (EA) of FMDV, and build up disinfection stations in the EA to carry out disinfection and control measures. Subsequently, the local ADC should carry out epidemiological investigation, monitoring and risk evaluation of susceptible animals in EA. Veterinarian should urgently carry out booster immunization for susceptible animals using inactivated vaccines. Last but not least, the trade markets of susceptible animals in the EA should be closed.

To relieve the FMD alert: the provincial ADC and OIE/China National Foot-and-Mouth Disease Reference Laboratory are responsible for FMD surveillance since the infection occurred. If no new cases of infected animals occur within 14 days of the slaughter of the last infected animal in the EA, the local veterinary bureau should apply to the local government to announce the relieving of the FMD alert in the EA. The animal or animal product circulation should resume from the EA three months after the FMD alert was lifted.

The Chinese government has conducted a long-term plan for FMD control and prevention. The objective of the plan is to achieve FMD serotype Asia 1 and serotype A free status in the whole China region without vaccination by 2020.

### Routine laboratory diagnostic techniques for FMDV

#### Isolation of FMDV viruses

As requested by OIE/China National Foot-and-Mouth Disease Reference Laboratory, a sample of at least 1 g of epithelial tissue (from the tongue, buccal mucosa or feet), homogenized in a transport medium composed of 5 times the amount of 0.04 M phosphate buffer (pH 7.2–7.6) and incubated overnight at 4°C should be sent to the laboratory. This FMDV suspect fluid is then centrifuged at 10,000 rpm for 10 min and 0.2 ml of this supernatant is injected into 10 2–5 day old unweaned mice to grow the virus. Some field viruses may require several passages before they become adapted to mice [3]. Meanwhile, the field suspected FMDV samples are also inoculated onto bovine thyroid cell or BHK-21 (baby hamster kidney) cell cultures for 48 hours to look for cytopathic effect (CPE). If no CPE is detected, the cells should be frozen and thawed and used to inoculate fresh cultures for the examination of CPE after another 48 hours [4]. Virus isolation in cell culture is considered to be the “gold standard” however, it may take up to four days to confirm which will delay the initiation of control procedures.

#### ELISA for FMDV diagnosis/typing

Virus isolation in primary cultures requires days/weeks (cell passages) before the results are obtained [5]. In general, the ELISA using type-specific serological reagents is the preferred procedure for the detection of FMD viral antigen and identification of viral serotype in the early stages of research because it is more specific, sensitive and efficient than virus isolation [6-8]. Different ELISA formats, particularly indirect ELISA, involved in blocking- or competition- or sandwich-based assays have been playing an increasing important role in the detection of FMDV or serological surveillance [9].

So far, over the past several decades, a series of ELISA methods have been developed in China for the diagnosis of FMD. These methods are theoretically consistent with Office International des Epizooties standards for FMD diagnosis and include antigen-capture ELISA for FMDV viral antigen typing, liquid-phase blocking ELISA (LPBE) for detection of antibodies against O, A and Asia 1 FMDV, and an indirect ELISA for detection of antibodies against the non-structural protein (NSP) 3ABC (3ABC-I-ELISA), which have been developed into diagnostic kits for FMDV diagnosis, serological surveillance and differentiation of FMD from swine vesicular disease viruses(SVD) [10].

LPBE for detection of antibodies against FMDV serotype A have been applied practically in 2010. This method replaces indirect haemagglutination and is a widely used assay for immune status surveys. The correlation between LPBE titer and challenge protection rate of animals immunized with FMD vaccine was established by challenging vaccinated animals with high virulent FMD virus [10].

The 3ABC ELISA (3ABC-I-ELISA) has been developed into a diagnostic kit for FMDV at the OIE/China National Foot-and-mouth Disease Reference Laboratory and applied in the field to differentiate between FMDV infection and vaccination. This can be used as a reliable tool for massive serological surveys to estimate the level of subclinical virus infection and to detect early incursion of FMDV into an animal population, regardless of viral serotype involved. These tests are especially important in the vaccination scenario due to its effectiveness on large-scale farm evaluation [10]. The shortcoming of the method is false-positive results for the sera of boars or bulls, which are subjected to multiple immunizations with inactivated vaccine [11].

#### RT-PCR and multiplex RT-PCR or real-time PCR

Compared to the virus isolation and antigen-capture ELISA, reverse transcription PCR (RT-PCR) has the advantage of greater sensitivity, reproducibility, reduced risk of carry-over contamination, and the results can be obtained in about 2 to 4 hours. RT-PCR can also be used to amplify genome fragments of FMDV of diagnostic materials including epithelium, milk, serum and OP samples. OIE/China National Foot-and-mouth Disease Reference Laboratory provides two RT-PCR kits for FMDV diagnosis, including general RT-PCR and a multiplex RT-PCR (mRT-PCR) kit. The real-time RT–PCR kit was designed to detect a 91 base pair nucleotide fragment within the 3D coding region. This method is sensitive enough to detect 0.1 TCID_50_ of FMDV from cell culture samples [10].

In OIE/China National Foot-and-mouth Disease Reference Laboratory, the multiplex RT-PCR is the preferred technology for investigation of FMDV. Three different fragments are amplified in this multiplex RT-PCR, 634 bp, 483 bp and 278 bp, representing FMDV serotypes A, Asia 1 and O samples, respectively. In the assay, three primer sets were used in a multiplex RT-PCR for rapid detection of virus in tissue samples or OP fluids of carriers of FMD. By testing ten-fold serial dilutions of FMDV, the sensitivity of multiplex RT-PCR is higher than that of conventional RT-PCR. This method has the potential to detect the prevalence of FMDV, and differentiate between other related viruses (BVDV, SVDV, VSV, VESV) [12].

### New detection methods

The characterization and limitation of new diagnostic methods including lateral flow device, the new ELISA assay, RT-LAMP and immuno-PCR assays for FMDV were reviewed, respectively. The lateral flow device has been validated and the other assays were being developed or in the process of validation in China.

#### Lateral flow device (LFD)

Ferris et al., developed a lateral flow device (LFD) for the detection of FMDV using different FMDV Mab [13]. The sensitivity and specificity of the LFD for FMDV was compared to ELISA. The results indicated that the sensitivity for the detection of FMDV serotype SAT2 was enhanced from 65% to 90% when the monoclonal antibody (Mab) 1 F10 in the devices was substituted with the Mab 2H6. The specificity of the LFD (99.4%) is comparable and the sensitivity (88.2%) for the detection of FMDV type SAT 2 antigen is superior to that of the slower and more complicated antigen ELISA (100% and 79%). The sensitivity and specificity of the LFD assay relies on the recognition of the Mab to the conservative antigenic epitope of FMDV. Compared to ELISA, the LFD procedure is very simple, rapid, and easy to perform and the results can be read by the naked eye. The LFD has the potential to be used in the pen-side diagnosis and serotyping of FMD [13,14]. The official veterinarian can start to implement measures according to the result of LFD as preliminary proof of an outbreak.

### New ELISA assay

A serotype-specific Mab-based antigen detection ELISA developed at the Istituto Zooprofilatico Sperimentale, Brescia, Italy, relies on a mixture of at least 3 different Mabs against each of the serotypes O, A and C to detect FMD virus in clinical samples [15].

As the monoclonal antibodies (Mabs) of FMDV define a specific region, a large number of viruses can be analyzed against a panel of Mab in a single test [16]. In our laboratory, we obtained the MAbs of FMDV with the exact epitope that covers the region from amino acid 133 to 160 of VP1 of FMDV Serotype O (CHA/1999). We developed the Mab based antigen capture ELISA for the detection of FMDV type O, which could discriminate between serotype A, C, Asia-1 and SVDV, so the development of synthetic technologies for conformational peptides would have great potential for preparation of monoclonal antibodies against conformational epitopes [17]. The MAbs with the exact epitopes could also be used to develop the assays for the specific FMDV field viruses.

### RT-LAMP

Conventional laboratory diagnosis of FMD is conducted with ELISA detection of specific viral antigens and by observation of cytopathic effects in cell culture [18]. Alternatively, the conventional reverse transcriptase polymerase chain reaction (RT-PCR) [19] and real-time RT-PCR [20] were used to complement primary diagnosis of the FMDV infection. These assays were laborious and required relatively expensive laboratory facilities. To solve these problems, a rapid, simple and practical assay, designated reverse transcription loop-mediated isothermal amplification (RT-LAMP), was developed to detect FMDV in animal and its products.

**Figure 1:**
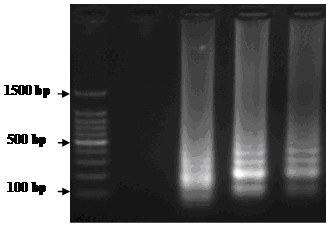
**Agarose gel electrophoresis analysis of RT-LAMP products using FMDV reference strains.** Lane M, DNA 100 bp Marker; Lane 1, PRRSV; Lane 2, O/CHA/1999; Lane 3, A/CHA/2009; Lane 4, Asia 1/CHA/2005.

The RT-LAMP assay was firstly reported by Notomi et al., [21] and applied successfully to the detection of many animal viruses [22-24]. The RT-LAMP reaction result can be detected on a 2.5% agarose gel electrophoresis with the addition of SYBR Green I to increase the ease of detection by the naked eye [25]. Our laboratory has evaluated the potential of RT-LAMP for detection of FMDV RNA. As shown in Figure 1, the agarose gel electrophoresis analysis of RT-LAMP products using FMDV reference strains was conducted. Compared to RT-PCR, the detection sensitivity of FMDV RT-LAMP was greater than RT-PCR using the same templates at identical concentrations. The result indicated that the detection limit of FMDV RT-LAMP was 10 copies, whereas RT-PCR was 100 copies per reaction [26].

To assess the specificity of RT-LAMP, cross reactivity with RNA from CSFV, SVDV, PRRSV and JEV was examined. The results indicated that the FMDV RT-LAMP assay did not detect CSFV, SVDV, PRRSV and JEV, as well as gave a negative reaction with tissues of healthy swine. FMDV RNA gave a positive reaction [26].

RT-LAMP is a sensitive diagnostic method, which is quite simple, requiring only a conventional water bath or heat block for incubation under isothermal conditions [22-24]. Another useful feature of RT-LAMP is that its products can be observed directly by the naked eye, because a white precipitate of magnesium pyrophosphate forms in the reaction tube [27]. The RT-LAMP assay is a timesaving procedure, as well, since the results can be obtained within 1 hour, whereas RT-PCR method typically requires 2 to 4 hours. The success of the LAMP assay depends on the high match between the four or six primers with the target sequence of FMDV. The characteristics of quasi-species of FMDV may reduce the sensitivity of the RT-LAMP assay, therefore it is necessary to evaluate the sensitivity of the assay using a large quantity of field samples of FMDV before practical application. Furthermore, the RT-LAMP assay has the potential for pen-side diagnosis as a rapid diagnostic assay, if it can be developed to work directly with clinical samples include epithelial tissue, OP fluid etc., rather than extracted RNA. The sensitivity and specificity of pen-side FMDV RT-LAMP should be evaluated in the future.

### GNP-IPCR

Recently, a new highly sensitive assay, namely a bio-barcode amplification (BCA) assay, was developed for ultrasensitive detection of target proteins and nucleic acids, with the detection limit reaching the level of 30 attomolar (aM) [28]. This method has been applied successfully to the detection of many animal viruses [29-31] and offers an excellent analytical sensitivity compared ELISA and conventional PCR. Based on the principles of the bio-barcode assay, a highly sensitive gold nanopariticle (GNP) improved immuno-PCR (GNP-IPCR) assay for the detection of FMDV antigen was designed. The target viral particles were captured by polyclonal or monoclonal antibodies coated to ELISA microplates, followed by incubation with GNP, which had been dually modified with oligonucleotides and a FMDV specific monoclonal antibody. After the formation of an immune complex and several washing steps, the signal DNA was released by heating and assayed by PCR.

**Figure 2:**
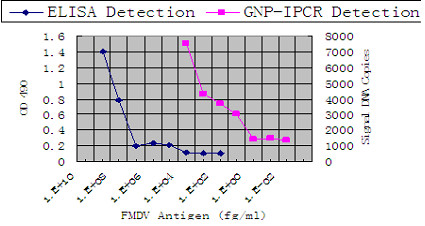
**Detection of serial dilutions of purified FMDV using GNP-IPCR and ELISA.** The detect limit of ELISA was 100 ng/ml purified FMDV and that of GNP-IPCR was 10 fg/ml.

To examine the sensitivity of GNP-IPCR and ELISA, we applied both methods to detect purified FMDV antigens. As shown in Figure 2, the detection limit of ELISA is 100 ng/ml, but the detection limit of GNP-IPCR is about 10 fg/ml of FMDV purified antigens, which is 7 titers of magnitude more sensitive than the general ELISA [29].

Although this method combining the applicability of detection of antigen from ELISA and the high sensitivity of PCR which offers an excellent analytical sensitivity due to very high signal amplification, the specificity was easily affected if the washing step was not performed thououghly. Nevertheless, GNP-IPCR is still an improved assay compared with immuno-PCR.

## Conclusion

Foot-and-mouth disease virus (FMDV) is a severe pathogen which can cause widespread epidemics. Early detection of FMD is essential for effective control of the disease. A simple, rapid, noninvasive diagnostic test is critical for FMD diagnosis. Although many diagnostic assays or kits are available to date for the control of FMDV, many works including an efficient veterinary disease prevention system, rapid diagnostic tests and an efficacious vaccine are still urgently needed with regard to FMD eradication.

## Competing interests

The authors declare that they have no competing interests.

## Authors’ contributions

YZD contributed to the original draft of the manuscript, and approved the final version. LZ, YXG, JZ and HTC contributed to conception and involved in revising the manuscript. JHZ, LNM and YSL helped to provide information and suggestion. All authors read and approved the final manuscript.
